# Risk of Autoimmune Diseases Following Optic Neuritis: A Nationwide Population-Based Cohort Study

**DOI:** 10.3389/fmed.2022.903608

**Published:** 2022-06-13

**Authors:** Kevin Sheng-Kai Ma, Chee-Ming Lee, Po-Hung Chen, Yan Yang, Yi Wei Dong, Yu-Hsun Wang, James Cheng-Chung Wei, Wen Jie Zheng

**Affiliations:** ^1^Department of Pediatrics, The Second Affiliated Hospital and Yuying Children’s Hospital of Wenzhou Medical University, Wenzhou, China; ^2^Center for Global Health, Perelman School of Medicine, University of Pennsylvania, Philadelphia, PA, United States; ^3^Graduate Institute of Biomedical Electronics and Bioinformatics, College of Electrical Engineering and Computer Science, National Taiwan University, Taipei, Taiwan; ^4^Department of Epidemiology, Harvard T.H. Chan School of Public Health, Boston, MA, United States; ^5^Department of Ophthalmology, Jen-Ai Hospital, Taichung, Taiwan; ^6^Institute of Medicine, Chung Shan Medical University, Taichung, Taiwan; ^7^Department of Health and Leisure Management, Yuanpei University of Medical Technology, Hsinchu, Taiwan; ^8^Department of Ultrasound, The Second Affiliated Hospital and Yuying Children’s Hospital of Wenzhou Medical University, Wenzhou, China; ^9^Department of Medical Research, Chung Shan Medical University Hospital, Taichung, Taiwan; ^10^Department of Allergy, Immunology and Rheumatology, Chung Shan Medical University Hospital, Taichung, Taiwan; ^11^Graduate Institute of Integrated Medicine, China Medical University, Taichung, Taiwan

**Keywords:** cohort study, ankylosing spondylitis, rheumatoid arthritis, systemic lupus erythematosus, psoriatic arthritis, myasthenia gravis, autoimmune diseases, optic neuritis

## Abstract

**Objectives:**

Optic neuritis is (ON) is believed to be an immune-mediated disease; however, the association between optic neuritis and autoimmune diseases remains unclear. This study aimed to identify the incidence rate and adjusted hazard ratio (aHR) of autoimmune diseases in patients with optic neuritis.

**Methods:**

This nationwide, population-based, retrospective cohort study collected patients’ data between 1999 and 2013 from the National Health Insurance Research Database in Taiwan. A total of 9,235 patients were included. Using 1:4 propensity scoring, 1,847 patients were enrolled in the optic neuritis group and 7,388 in the non-optic neuritis group according to age, sex, comorbidities, and corticosteroid use. Follow-up was started from the index date and the endpoint was a diagnosis of new-onset autoimmune diseases including, myasthenia gravis (MG), psoriatic arthritis (PsA), systemic lupus erythematosus (SLE), rheumatoid arthritis (RA), and ankylosing spondylitis (AS).

**Results:**

The Kaplan-Meier curves depicted that patients with optic neuritis had a higher cumulative incidence of autoimmune diseases than patients without optic neuritis. Cox proportional hazard regression showed that patients with optic neuritis were at a high risk of autoimmune diseases (aHR: 1.40; 95% C.I., 1.05–1.87), including MG (aHR: 4.16, 95% C.I.: 1.33–12.94), SLE (aHR: 3.33, 95% C.I.: 1.24–8.97), and AS (aHR: 2.86, 95% C.I.: 1.54–5.31). Subgroup analysis provided that patients with optic neuritis aged below 65 years (aHR: 1.42, 95% C.I.: 1.03–1.96) or who were females (aHR: 1.59, 95% C.I.: 1.11–2.27) had a significantly increased risk of autoimmune diseases compared to respective controls. The use of corticosteroids reduced the risk of autoimmune diseases in patients with optic neuritis (aHR for corticosteroids non-users: 1.46, 95% C.I.: 1.03–2.07).

**Conclusion:**

Patients with optic neuritis presented with a high risk of autoimmune diseases such as MG, SLE, and AS, especially patients with optic neuritis who were young or females. Corticosteroids attenuated the link between optic neuritis and subsequent autoimmune diseases.

## Introduction

Optic neuritis is a demyelinating optic neuropathy affecting one or both optic nerves. The incidence of optic neuritis ranges from 0.83 to 5.36 per 100,000 (1–3), with its pathophysiology remaining unclear. It is believed to be an immune-mediated disease, supported by the identification of systemic T-cells at disease onset and B-cells against myelin basic protein in the cerebrospinal fluid (CSF) of patients with optic neuritis (4). Optic neuritis is clinically divided into typical and atypical forms. Typical optic neuritis is a demyelinating clinically isolated syndrome generally associated with multiple sclerosis, while atypical optic neuritis can be classified into those with or without systemic disease association. Atypical optic neuritis without systemic disease included neuromyelitis optica spectrum disease (NMOSD), myelin oligodendrocyte glycoprotein (MOG) optic neuritis, and chronic relapsing inflammatory optic neuropathy (CRION) (1–3). Lastly, atypical optic neuritis associated with systemic diseases can present with sarcoidosis, connective tissue diseases, and vasculitis. Association between optic neuritis and immune-mediated inflammatory diseases such as psoriasis and Crohn’s disease has also been reported in an epidemiologic study of optic neuritis (3).

Although the pathophysiologic mechanism remains ambiguous, several anatomical features may be related to optic neuritis. The limited space of the optic canal makes the optic nerve susceptible to compression when swelling. Nerves were profoundly affected by compression. Besides, the inflammatory cells can easily infiltrate through the subarachnoid space, pia, and pial mater around the optic nerve (1–3). Moreover, the intracranial subarachnoid space is connected with the orbital subarachnoid space, for which intracranial inflammation can affect the optic nerve due to the cul-de-sac anatomy of the optic nerve. Permeability of the prelaminar optic nerve head also results in the lack of classical blood-brain barrier features (1–3).

Since autoimmune diseases are mainly characterized by T cell dysregulation and B cell response against self-antigens in tissues or organs (5) with distinct and heterogeneous clinical manifestations (6), it is possible that optic neuritis can be either an early sign of or a risk factor for autoimmune diseases and subsequent systemic involvement. As such, this study aimed to ascertain the association between optic neuritis and autoimmune diseases, with particular focus on myasthenia gravis (MG), rheumatoid arthritis (RA), ankylosing spondylitis (AS), psoriatic arthritis (PsA), and systemic lupus erythematosus (SLE) with a population-based cohort registry.

## Materials and Methods

### Data Source

This nationwide, population-based, retrospective, cohort study used data from the Longitudinal Health Insurance Database (LHID), which includes 1 million people randomly sampled from the National Health Insurance Research Database (NHIRD) to represent over 99% of 23 million population in Taiwan. Claims data from January 1, 2000 to December 31, 2012 were used as the data source. The study was approved by the Institutional Review Board of Chung Shan Medical University, Taiwan, R.O.C. (number CS15134).

### Study Group and Outcome Measurement

The study population included patients with optic neuritis in LHID from January 1, 2000 to December 31, 2012 (*n* = 2031). Diagnosis of optic neuritis was based on typical symptoms of the patient, including orbital pain while moving the eyes, acute onset of visual loss, decreased visual acuity, and defect of the visual field. A magnetic resonance imaging (MRI) scan was performed for patients with monosymptom or clinically isolated syndrome to confirm the diagnosis. Optic neuritis was identified as either isolated optic neuritis, optic neuritis with association of multiple sclerosis, or antibody-related optic neuritis such as NMOSD typically with aquaporin-4 antibody (AQP4-Ab) seropositivity and simultaneous immune-mediated myelitis, and MOG optic neuritis, typically with myelin oligodendrocyte glycoprotein seropositivity, bilateral optic disk edema, and markedly steroid responsive (7–9). A new diagnosis of optic neuritis was defined by at least two outpatient visits or one admission from the database. The index date was set for the date of the first optic neuritis diagnosis. Patients diagnosed with optic neuritis before the index date were excluded. The definition of a new diagnosis of autoimmune diseases in our study involved the above-mentioned MG, PsA, SLE, RA, and ankylosing spondylitis (AS). Physical examinations, blood tests, and imaging were performed to diagnose autoimmune diseases. MG was diagnosed by clinical symptoms such as myasthenic weakness and validated by seropositivity of antibodies against the acetylcholine receptor (AChR) and muscle-specific kinase (MuSK); electromyography stimulation was used in seronegative cases but highly suspected MG patients (10). According to 2019 European League Against Rheumatism (EULAR)/American College of Rheumatology (ACR) Classification Criteria for SLE, diagnosis of SLE was confirmed by indirect immunofluorescence assay (IFA) with antinuclear antibodies (ANA) at a titer of ≥1:80 on HEp-2 cells; additive criteria were counted to ensure that the patient has at least one clinical symptom with a total score ≥ 10 (11). According to the 2010 ACR/EULAR RA Classification Criteria, definite RA diagnosis was made when patients had synovitis and their symptoms or serological tests outcomes met with the criteria, which included the high numbers of joint involvement, rheumatoid factor (RF), and anti-citrullinated protein antibody (ACPA) seropositivity, abnormal C-reactive protein (CRP) and erythrocyte sedimentation rate (ESR), with a duration of symptoms ≥ 6 weeks and addition of score ≥ 6/10 (12). Eventually, PsA and AS are both spondyloarthropathies (SpA). According to the Assessment of Spondyloarthritis International Society (ASAS) criteria for axial spondyloarthritis (axSpA), patients who suffered from back pain for more than 3 months and the age at onset was less than 45 years old were considered as suspected AS. The diagnosis was confirmed with one image finding of sacroiliitis plus at least one SpA symptom, or human leukocyte antigen (HLA)-B27 positivity plus at least 2 SpA features (13, 14). In addition, the diagnosis of PsA was based on Classification Criteria for Psoriatic Arthritis (CASPAR). Patients with inflammatory articular disease plus scores addition ≥ 3 according to certain criteria including psoriasis, nail dystrophy, dactylitis, and radiographic findings (13, 15) will be diagnosed with PsA. New diagnoses of autoimmune diseases were defined by at least two outpatient visits or one admission as well. Patients diagnosed with the above-mentioned autoimmune diseases before the index date were excluded (*n* = 165). The follow-up period was defined as the duration from the index date to the date of a newly diagnosed autoimmune diseases, withdrawal from insurance, or December 31, 2013, whichever came first.

### Covariates and Matching

The comparison non-optic neuritis cohort was matched with the optic neuritis cohort at a ratio of 1:8 according to age and sex. To ensure baseline comparability, the non-optic neuritis group was matched with the optic neuritis group by age, sex, and comorbidities. The presence of comorbidities was defined as a diagnosis of hypertension, hyperlipidemia, chronic liver disease, chronic kidney disease, diabetes, cardiovascular diseases (16), malignancy, chronic obstructive pulmonary disease (COPD), ischemic heart disease, stroke, and the use of corticosteroid by performing a matching ratio 1:4 of propensity score (study period usage ≥ 30 days). In our study, the comorbidities mentioned above must be diagnosed 1 year before the index date and were based on at least two outpatient visits or one admission used in the previous studies (17–25).

### Statistical Analysis

The comparison between the optic neuritis and non-optic neuritis groups was performed by absolute standardized differences (ASD). ASD < 0.1 indicated the similarities of the characteristics in both groups. The cumulative incidences of autoimmune diseases in both groups were plotted with Kaplan–Meier methods, and a log-rank test was performed to test the significance. The hazard ratio (HR) for autoimmune diseases was evaluated by the Cox proportional hazard model. SPSS version 18.0 was used to analyze all statistical data. A *p*-value < 0.05 was considered statistically significant.

## Results

### Study Population

**FIGURE 1:**
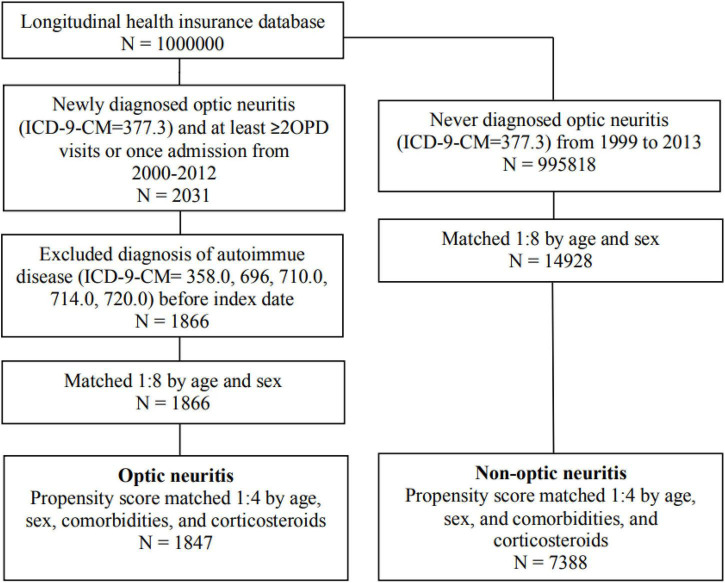
Flowchart of the study design.

Figure 1 illustrated the sample selection process flowchart. Initially, 2031 patients with newly-diagnosed optic neuritis during the study period were identified and included in the optic neuritis group. Among them, 165 patients who had been diagnosed with autoimmune diseases before the index date were excluded. The non-optic neuritis group comprised 995818 individuals who were never diagnosed with optic neuritis between 1999 and 2013. 14928 controls were matched with the optic neuritis cohort at a ratio of 1:8 based on age and sex. The final cohort consisted of 1847 patients in the optic neuritis group and 7388 controls in the non-optic neuritis group.

### Baseline Characteristics of Patients With Optic Neuritis and Non-optic Neuritis Controls

In the optic neuritis group, 51.4% were women and 48.6% were men. Before propensity score matching, prominent differences were observed between two distributions, chronic liver disease, and corticosteroid use, in the optic neuritis group than the non-optic neuritis group (ASD > 0.10). However, after the propensity score had been calculated, there were no differences in the baseline characteristics and comorbidities between the two groups (ASD < 0.10) (Table 1).

**TABLE 1:** Demographic characteristics of optic neuritis group and non-optic neuritis group.

	Matched by age and sex			After PSM		
	Optic neuritis (*N* = 1866)	Non-optic neuritis (*N* = 14928)	ASD	Optic neuritis (*N* = 1847)	Non-optic neuritis (*N* = 7388)	ASD
Age			<0.001			0.030
<20	128 (6.9)	1024 (6.9)		126 (6.8)	547 (7.4)	
20–39	390 (20.9)	3120 (20.9)		388 (21.0)	1510 (20.4)	
40–64	911 (48.8)	7288 (48.8)		903 (48.9)	3600 (48.7)	
≥65	437 (23.4)	3496 (23.4)		430 (23.3)	1731 (23.4)	
Mean ± SD	50.3 ± 18.4	50.3 ± 18.4	<0.001	50.3 ± 18.4	50.0 ± 18.4	0.013
Sex			<0.001			0.021
Female	959 (51.4)	7672 (51.4)		952 (51.5)	3885 (52.6)	
Male	907 (48.6)	7256 (48.6)		895 (48.5)	3503 (47.4)	
Hypertension	416 (22.3)	2862 (19.2)	0.077	405 (21.9)	1431 (19.4)	0.063
Hyperlipidemia	142 (7.6)	1043 (7.0)	0.024	138 (7.5)	452 (6.1)	0.054
Chronic liver disease	123 (6.6)	617 (4.1)	0.109	111 (6.0)	413 (5.6)	0.018
Chronic kidney disease	34 (1.8)	131 (0.9)	0.082	26 (1.4)	101 (1.4)	0.003
Diabetes	196 (10.5)	1287 (8.6)	0.064	193 (10.4)	622 (8.4)	0.070
COPD	101 (5.4)	550 (3.7)	0.083	94 (5.1)	356 (4.8)	0.012
Cancer	73 (3.9)	354 (2.4)	0.088	66 (3.6)	249 (3.4)	0.011
Ischemic heart disease	127 (6.8)	821 (5.5)	0.054	121 (6.6)	403 (5.5)	0.046
Stroke	108 (5.8)	568 (3.8)	0.093	103 (5.6)	383 (5.2)	0.017
Corticosteroids	620 (33.2)	3017 (20.2)	0.297	601 (32.5)	2423 (32.8)	0.005

*ASD, absolute standardized differences; COPD, chronic obstructive pulmonary disease; PSM, propensity score matching.*

### Incidence of Autoimmune Diseases in Patients With Optic Neuritis

**FIGURE 2:**
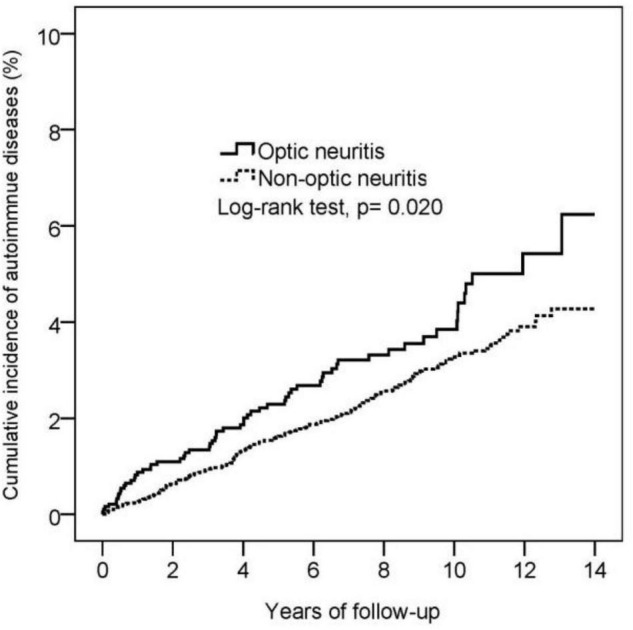
Kaplan-Meier curve for the cumulative incidence of autoimmune diseases in the optic neuritis group and non-optic neuritis group.

During the 13-year follow-up period, 62 patients with optic neuritis and 188 propensity score-matched controls without optic neuritis were diagnosed with autoimmune diseases. The incidence rate was 4.55 (95% C.I.: 3.55–5.84) and 3.24 (95% confidence C.I.: 2.81–3.74) per 1,000 person-years in patients with and without optic neuritis, respectively. Poisson regression showed that patients with optic neuritis had a higher relative risk (RR: 1.40, 95% CI: 1.05–1.87) for autoimmune diseases compared to patients without optic neuritis (Table 2). Furthermore, the cumulative incidence of autoimmune diseases in Kaplan–Meier curves indicated that patients with optic neuritis had a greater risk of autoimmune diseases than those without optic neuritis (log-rank, *p* = 0.02) (Figure 2).

**TABLE 2:** Poisson regression for the incidence and relative risk of autoimmune diseases in the optic neuritis group and non-optic neuritis group.

	Matched by age and sex		After PSM	
	Non-optic neuritis	Optic neuritis	Non-optic neuritis	Optic neuritis
*N*	14,928	1,866	7,388	1,847
Person-years	112,669	13,743	57,940	13,619
No. of autoimmune diseases	388	62	188	62
ID (95% C.I.)	3.44 (3.12–3.80)	4.51 (3.52–5.79)	3.24 (2.81–3.74)	4.55 (3.55–5.84)
Relative risk (95% C.I.)	Reference	1.31 (1.00–1.71)	Reference	1.40 (1.05–1.87)

*ID, incidence density (per 1000 person-years); PSM, propensity score matching.*

### Risk of Autoimmune Disease Following Optic Neuritis

The Cox proportional hazard regression illustrated that patients with optic neuritis had a higher risk for autoimmune diseases than patients without optic neuritis (adjusted HR, 1.40; 95% CI, 1.05–1.87). Generally, females had a higher risk of developing autoimmune diseases compared to males (adjusted HR for males: 0.7, 95% C.I.: 0.54–0.90). Patients aged between 40 and 64 years (adjusted HR: 2.13, 95% C.I.: 1.14–3.96) and patients with comorbid chronic liver disease (adjusted HR: 1.73, 95% C.I.: 1.10–2.70) were at a significantly high risk of autoimmune diseases (Table 3).

**TABLE 3:** Cox proportional hazard model for the risk of autoimmune diseases.

	Matched by age and sex		After PSM	
	HR^†^ (95% C.I.)	*p* value	HR^†^ (95% C.I.)	*p* value
**Group**				
Non-Optic neuritis	Reference		Reference	
Optic neuritis	1.31 (1.00–1.72)	0.047	1.40 (1.05–1.87)	0.021
**Age**				
<20	Reference		Reference	
20–39	1.04 (0.65–1.67)	0.862	1.41 (0.73–2.74)	0.306
40–64	1.71 (1.11–2.63)	0.015	2.13 (1.14–3.96)	0.017
≥65	1.56 (0.97–2.51)	0.069	1.91 (0.97–3.78)	0.062
**Sex**				
Female	Reference		Reference	
Male	0.69 (0.57–0.83)	<0.001	0.70 (0.54–0.90)	0.006
Hypertension	1.22 (0.93–1.59)	0.152	1.04 (0.72–1.51)	0.821
Hyperlipidemia	1.00 (0.68–1.47)	0.999	0.70 (0.38–1.29)	0.248
Chronic liver disease	1.49 (1.01–2.19)	0.042	1.73 (1.10–2.70)	0.017
Chronic kidney disease	0.86 (0.27–2.70)	0.797	1.32 (0.42–4.17)	0.637
Diabetes	0.93 (0.65–1.34)	0.712	1.02 (0.62–1.66)	0.945
COPD	1.37 (0.86–2.19)	0.180	1.24 (0.68–2.27)	0.477
Cancer	1.06 (0.56–2.00)	0.851	0.90 (0.40–2.04)	0.803
Ischemic heart disease	0.86 (0.55–1.34)	0.507	0.78 (0.41–1.48)	0.452
Stroke	1.39 (0.88–2.18)	0.157	1.50 (0.86–2.63)	0.155
Corticosteroids	0.78 (0.62–0.98)	0.033	0.88 (0.67–1.15)	0.360

*^†^Adjusted for age, sex, hypertension, hyperlipidemia, chronic liver disease, chronic kidney disease, diabetes, COPD, cancer, ischemic heart disease, stroke, and corticosteroids. PSM, propensity score matching.*

Subgroup analysis revealed the subpopulations of patients with optic neuritis that were more susceptible to autoimmune diseases, in which patients with optic neuritis patients aged below 65 years (adjusted HR: 1.42, 95% C.I.: 1.03–1.96) or who were females (adjusted HR: 1.59, 95% C.I.: 1.11–2.27) were prone to autoimmune diseases. Moreover, the use of corticosteroids had a protective effect on autoimmune diseases in patients with optic neuritis (adjusted HR: 1.46, 95% CI: 1.03–2.07) (Table 4). Sub-outcome analysis further provided that autoimmune diseases associated with optic neuritis included MG (adjusted HR: 4.16, 95% C.I.: 1.33–12.94), SLE (adjusted HR: 3.33, 95% C.I.: 1.24–8.97), and AS (adjusted HR: 2.86, 95% C.I.: 1.54–5.31), while the risk of PsA and RA following optic neuritis did not reach statistical significance (Table 5).

**TABLE 4:** Subgroup analysis of the association between optic neuritis and autoimmune diseases.

	Optic neuritis		Non-optic neuritis			
	*N*	No. of autoimmune diseases	*N*	No. of autoimmune diseases	HR^†^ (95% C.I.)	*p* value
**Age**						
<65	1417	50	5657	149	1.42 (1.03–1.96)	0.033
≥65	430	12	1731	39	1.31 (0.68–2.51)	0.413
						*p* for interaction = 0.837
**Gender**						
Female	952	41	3885	113	1.59 (1.11–2.27)	0.011
Male	895	21	3503	75	1.16 (0.72–1.89)	0.539
						*p* for interaction = 0.339
**Corticosteroids**						
No	1246	43	4965	121	1.46 (1.03–2.07)	0.034
Yes	601	19	2423	67	1.27 (0.76–2.13)	0.354
						*p* for interaction = 0.614

*^†^Adjusted for all variables.*

**TABLE 5:** Sub-outcome analysis of the association between optic neuritis and autoimmune diseases.

	Optic neuritis		Non-optic neuritis			
	*N*	No. of autoimmune diseases	*N*	No. of autoimmune diseases	HR (95% C.I.)	*p* value
Myasthenia gravis*^a^*	1,847	6	7,388	6	4.16 (1.33–12.94)	0.014
Psoriatic arthritis*^b^*	1,847	10	7,388	63	0.67 (0.34–1.31)	0.242
System lupus erythematosus*^c^*	1,847	7	7,388	9	3.33 (1.24–8.97)	0.017
Rheumatoid arthritis*^d^*	1,847	22	7,388	88	1.06 (0.66–1.69)	0.804
Ankylosing spondylitis*^e^*	1,847	17	7,388	25	2.86 (1.54–5.31)	<0.001

*^a^Adjusted for age, sex, hypertension, hyperlipidemia, chronic liver disease, diabetes, COPD, cancer, ischemic heart disease, stroke, and corticosteroids.*

*^b^Adjusted for age, sex, hypertension, hyperlipidemia, chronic liver disease, diabetes, COPD, ischemic heart disease, stroke, and corticosteroids.*

*^c^Adjusted for age, sex, hypertension, hyperlipidemia, chronic liver disease, stroke, and corticosteroids.*

*^d^Adjusted for age, sex, hypertension, hyperlipidemia, chronic liver disease, chronic kidney disease, diabetes, COPD, cancer, ischemic heart disease, stroke, and corticosteroids.*

*^e^Adjusted for age, sex, hypertension, hyperlipidemia, chronic liver disease, chronic kidney disease, diabetes, cancer, ischemic heart disease, stroke, and corticosteroids.*

## Discussion

Optic neuritis has been recently considered as an immune-mediated diseases with speculated autoimmune pathogenesis, as evidenced by studies providing that seropositive autoantibodies subtypes including myelin oligodendrocyte glycoprotein antibody (MOG-Ab) and seropositive aquaporin-4 antibody (AQP4-Ab) (26) typically present in patients with optic neuritis. In the present cohort study, it was demonstrated that optic neuritis was followed by a significantly great risk of autoimmune diseases including MG, SLE, and AS, for which optic neuritis can be an early sign or independent risk factor for autoimmune diseases. Moreover, patients with optic neuritis aged below 65 years or those who were females were the most susceptible to optic neuritis-associated autoimmune disease, which was in accordance with previous studies showing that 55%–70% of optic neuritis occurs in women with a bigger proportion of young adults(2, 3).

As for the associations between optic neuritis and autoimmune diseases, cohort study in the United Kingdom (2) of 10,937,511 people suggested that patients with optic neuritis had significantly higher risks of Behçet disease, vasculitis, and Sjogren’s syndrome. This was consistent with our findings of optic neuritis patients with higher risks for autoimmune diseases compared with patients without optic neuritis, demonstrated by the Kaplan–Meier curve.

The estimated incidence rate of SLE varies from 1 to 25 per 100,000 person-years in North America, South America, Europe, and Asia with a predilection for Asians and Africans (27–30). In our study, we consistently suggested a high incidence rate of SLE (22.4 per 100,00 persons-year) in Taiwanese patients. Among all the subtypes of SLE, neuropsychiatric SLE (NPSLE) was the subtype that presented with stroke, seizures, altered mental status, cognitive impairment and other neurologic or psychiatric symptoms. More importantly, optic neuritis can even be the initial clinical manifestation of neuropsychiatric SLE (31). For the increased risk of SLE following optic neuritis, as provided in the sub-outcome analysis in the present study, it is possible that optic neuritis can be an indicator or early sign of neuropsychiatric SLE.

Predictor variables of autoimmune diseases included age, sex, and chronic liver diseases. Particularly, SLE is diagnosed at a mean age of 35 years, and RA typically occurs in middle-aged individuals (32–34). On the other hand, psoriasis and MG can present at any age; however, they are more common in adults than in children. Psoriasis has a bimodal distribution in age; the first peak occurs between 30-39 years and the second peak between 50 and 69 years (35, 36). In line with these previous reports, the Cox proportional hazard model in our study indicated a higher risk of developing autoimmune diseases in patients aged 40-64 years. Compared to men, women are at a two-fold and nine-fold risk for RA and SLE, respectively; however, no sex predominance has been observed in psoriasis (32–34). Despite the inclusion of AS, which was more commonly diagnosed in men, our study showed that males had a lower risk for autoimmune diseases compared to females. Previous cohort studies found that patients with PsA and RA were at an increased risk of liver diseases including cirrhosis and non-alcoholic fatty liver disease (NAFLD); particularly, PsA patients treated with systemic therapy had the greatest risk of NAFLD (37, 38). In our study, patients with optic neuritis were comorbid with chronic liver disease at baseline; moreover, chronic liver disease was an independent risk factor for autoimmune disease.

It is believed that optic neuritis is an immune-mediated disease triggered by inflammation and causes axon demyelinating injury. Optic nerve degeneration and visual loss are both pathological changes of optic neuritis (39). Activated systemic T cells, which trigger the release of cytokines and inflammatory mediators, play an important role in the acute phase 4. It is common to find increased autoimmune B cells against MOG-ab in CSF of patients with optic neuritis (40, 41) and patients with SLE. Furthermore, MG is an acquired autoimmune neuromuscular junction disorder disease that also involves the activation of T-cells and stimulation of B-cell antibody production, specifically, acetylcholine receptor antibodies and muscle-specific kinase antibodies (42). Although the underlying mechanism remains unclear, we believe that there may be a relationship between these autoimmune diseases. Our data showed that patients with optic neuritis had a higher HR for autoimmune diseases than patients without optic neuritis, especially autoimmune diseases such as MG, SLE, and AS. Among all patients with optic neuritis, those who were young and those who were females were at an especially high risk of developing autoimmune diseases. The finding on the use of corticosteroids attenuating the risk of autoimmune disease supported inflammation as the underlying mechanism that connected optic neuritis and autoimmune diseases.

There are several strengths in our study. First, this is a nationwide population-based study, in which a large sample size was (23, 43–45) qualified for an assessment of the relationship between optic neuritis and autoimmune diseases during a 14-year period. Second, this is the first nationwide population-based cohort study that demonstrated the significant association between optic neuritis and autoimmune diseases, in particular MG, SLE, and AS. Nevertheless, this study has certain limitations. First, ICD-9-CM codes were used to retrieve diagnoses of optic neuritis and autoimmune diseases, which may be biased. There is a risk of misdiagnosis or miscoding in the hospital, resulting in data bias. Second, the definition of autoimmune diseases encompassed only five diseases. Other autoimmune diseases such as vasculitis and Sjogren syndrome were not included in this study. Third, although neuropsychiatric SLE (NPSLE) may be the subtype that was most related to optic neuritis due to its neurologic involvement, we were not capable of distinguishing the subtypes among SLE patients because the database we utilized did not sort SLE patients by their subtypes. As the result, we were not able to carry out the comparison of the subgroup analysis of SLE. These limitations will be considered in our future works.

In conclusion, this cohort study elaborated on prominent risk of autoimmune disease in patients with optic neuritis, especially patients with optic neuritis who were young or females. Notably, the use of corticosteroids attenuated the link between optic neuritis and autoimmune diseases. Clinicians should consider this association as a guide in managing patients with optic neuritis. Further investigations with a longer follow-up period are necessary to ensure the relationship between optic neuritis and autoimmune diseases.

## Data Availability Statement

The original contributions presented in the study are included in the article/supplementary material, further inquiries can be directed to the corresponding authors.

## Ethics Statement

This study was approved by the Institutional Review Board of Chung Shan Medical University (number CS15134). Written informed consent for participation was not required for this study in accordance with the national legislation and the institutional requirements.

## Author Contributions

KM, C-ML, WZ, and P-HC participated in the research design and writing of the manuscript. KM, YY, and YD collected the data and wrote the original draft. KM and Y-HW participated in data collection and statistical analysis, and JW was a project administrator and revised the manuscript. All authors contributed to the study and have approved the final manuscript.

## Conflict of Interest

The authors declare that the research was conducted in the absence of any commercial or financial relationships that could be construed as a potential conflict of interest.

## Publisher’s Note

All claims expressed in this article are solely those of the authors and do not necessarily represent those of their affiliated organizations, or those of the publisher, the editors and the reviewers. Any product that may be evaluated in this article, or claim that may be made by its manufacturer, is not guaranteed or endorsed by the publisher.
